# Influence of Electricity on Epidemics

**Published:** 1866-07

**Authors:** 


INFLUENCE OF ELECTRICITY ON EPIDEMICS.

The next paper taken up was the report of a special committee on the Influence of Electricity on Epidemics, by Dr. Squire Littell, of Philadelphia.

The report was presented to the Section on Hygiene at the last session of the Association, but owing to its length was not all read. It was deferred for the time, and owing to the supposed absence of the author from the country, the paper was omitted from the Transactions. Members of the section present who had carefully examined the paper, spoke of it in high terms, and as being well worthy of publication in the Transactions. But as it was a long paper, and had already been examined, and in part at least discussed, they did not deem it worth while to again occupy the section with its consideration.

On motion, it was referred to the Committee on Publication. The section then adjourned.

*Second Session, May 2.*

